# Supplementary material for: Patterns of Intron Gain and Loss in Fungi
Source: PLoS Biol. 2004 Nov 30;2(12):e422. doi: 10.1371/journal.pbio.0020422 (PMC532390; doi:10.1371/journal.pbio.0020422)
Supplement: Table S1 — Also available at http://genes.mit.edu/NielsenEtAl/. (4.3 MB ZIP). [file pbio.0020422.st001.zip › NielsenEtAl/html/62.html]

AN9465.1.NCU02744.1.MG04829.1.FG01154.1


```
 CLUSTAL W (1.82) Multiple Sequence Alignments - Introns Inserted


Sequence 1: AN9465.1	192 aa
Sequence 2: FG01154.1	250 aa
Sequence 3: NCU02744.1	193 aa
Sequence 4: MG04829.1	194 aa
Alignment Length: 251 aa
Number Identitical Residues: 132 aa
Alignment Score (without introns) 5586


MG04829.1 	---------------------------------------------------------MKY
NCU02744.1	---------------------------------------------------------MRY
FG01154.1 	MASAVGCSFPTTFALTWKERALPLALTWNFSCPQIPKNSRTALDTLNPRHQHLTAGRMKY
AN9465.1  	---------------------------------------------------------MRY
          	                                                         *:*

MG04829.1 	IHSEELLEVPEGV1KISIKSRLITVEGPRG1KLTKSLNHIAVTFSQPSKNVIGIEIHHGK
NCU02744.1	IHSEETLEVPENV1KVSIKSRIVTVEGPRG~KLVKDLSHIAVNFSVIKKGVIGLEIHHGN
FG01154.1 	IHSQELLEIPEGV1KVAIKSRIVTVEGPRG~KLVKDLSHLAVNFTSTKKNQISIEIHHGV
AN9465.1  	IHSEETLPIPENV1KVHIRSRVVTVEGPRG1KLTKDLSHIAVTFGRPKKDVISIEMHHGS
          	***:* * :**.* *: *:**::******* **.*.*.*:**.*   .*. *.:*:*** 

MG04829.1 	RKDVATLRTVRTLINNLIIGVTKGYKYKMRYVYAHFPINVNVEKNNETGNAEVEIR2NFI
NCU02744.1	RKNVAALRTVRTIINNLIIGVTKGFKYKMRYVYAHFPINVNVEKNAETGNFEVEIR2NFI
FG01154.1 	RKNVATLRTVRTLINNLIIGVTKGFKYKMRYVYAHFPINVNVSQNSETDLYEVEIR~NFI
AN9465.1  	RKGVATLRTVRTLINNLIIGVTKGFKYKMRYVYAHFPINVNIEKNAETGQYDVEIR2NFL
          	**.**:******:***********:****************:.:* **.  :**** **:

MG04829.1 	GEKLVRRVAMQPGVEVEISKAQKDELILSGNSVEAVSQSAADIQQICKVRNKDIRK0FLD
NCU02744.1	GEKIVRRVVMQPGVDVEISKAQKDELVLSGNSLEGVSQSAADIQQICRVRNKDIRK0FLD
FG01154.1 	GEKLVRRIVMHPGVDVEASTTQKDELVLSGNSLENVSQSAADIQQICRVRNKDIRK0FLD
AN9465.1  	GEKYVRRVVCQPGVEIIASPNVKDELQLSGNSLEAVSQSAADIQQICRVRNKDIRK0FLD
          	*** ***:. :***::  *   **** *****:* ************:******** ***

MG04829.1 	GMYVSEKGNIEEIAA
NCU02744.1	GIYVSEKGNIVEDQ-
FG01154.1 	GLYVSEKGNVVQDE-
AN9465.1  	GLYVSEKGNIVEE--
          	*:*******: :
```
